# CD44 expression in the tumor periphery predicts the responsiveness to bevacizumab in the treatment of recurrent glioblastoma

**DOI:** 10.1002/cam4.3767

**Published:** 2021-02-05

**Authors:** Masahiro Nishikawa, Akihiro Inoue, Takanori Ohnishi, Hajime Yano, Yonehiro Kanemura, Shohei Kohno, Shiro Ohue, Saya Ozaki, Shirabe Matsumoto, Satoshi Suehiro, Yawara Nakamura, Seiji Shigekawa, Hideaki Watanabe, Riko Kitazawa, Junya Tanaka, Takeharu Kunieda

**Affiliations:** ^1^ Department of Neurosurgery Ehime University School of Medicine Toon Japan; ^2^ Department of Neurosurgery Washoukai Sadamoto Hospital Matsuyama Japan; ^3^ Department of Molecular and Cellular Physiology Ehime University School of Medicine Toon Japan; ^4^ Department of Biomedical Research and Innovation Institute for Clinical Research National Hospital Organization Osaka National Hospital Osaka Japan; ^5^ Department of Neurosurgery National Hospital Organization Osaka National Hospital Osaka Japan; ^6^ Department of Neurosurgery Ehime Prefectural Central Hospital Matsuyama Japan; ^7^ Division of Diagnostic Pathology Ehime University Hospital Toon Japan

**Keywords:** bevacizumab, CD44, glioblastoma, glioma stem cell, invasion, vascular endothelial growth factor

## Abstract

Antiangiogenic therapy with bevacizumab (Bev), a monoclonal antibody targeting vascular endothelial growth factor (VEGF), is a common treatment for recurrent glioblastoma (GBM), but its survival benefit is limited. Resistance to Bev is thought to be a major cause of ineffectiveness on Bev therapy. To optimize Bev therapy, identification of a predictive biomarker for responsiveness to Bev is required. Based on our previous study, we focused on the expression and functions of CD44 and VEGF in the Bev therapy. Here, we analyze a relationship between CD44 expression and responsiveness to Bev and elucidate the role of CD44 in anti‐VEGF therapy. CD44 and VEGF expression in the tumor core and periphery of 22 GBMs was examined, and the relationship between expression of these molecules and progression‐free time on Bev therapy was analyzed. The degree of CD44 expression in the tumor periphery was evaluated by the ratio of the mRNA expression in the tumor periphery to that in the tumor core (P/C ratio). VEGF expression was evaluated by the amount of the mRNA expression in the tumor periphery. To elucidate the roles of CD44 in the Bev therapy, in vitro and in vivo studies were performed using glioma stem‐like cells (GSCs) and a GSC‐transplanted mouse xenograft model, respectively. GBMs expressing high P/C ratio of CD44 were much more refractory to Bev than those expressing low P/C ratio of CD44, and the survival time of the former was much shorter than that of the latter. In vitro inhibition of VEGF with siRNA or Bev‐activated CD44 expression and increased invasion of GSCs. Bev showed no antitumor effects in mice transplanted with CD44‐overexpressing GSCs. The P/C ratio of CD44 expression may become a useful biomarker predicting responsiveness to Bev in GBM. CD44 reduces the antitumor effect of Bev, resulting in much more highly invasive tumors.

## Graphical abstract

In this research, we analyze a relationship between CD44 expression and responsiveness to Bev and elucidate the role of CD44 in anti‐VEGF therapy. These results indicate CD44 will be a useful biomarker for predicting responsiveness to bevacizumab and may serve as a therapeutic target in both primary and recurrent GBMs.

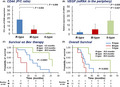

## 1 INTRODUCTION

Glioblastoma multiforme (GBM) is the most malignant primary brain tumor, with poor prognosis including a median survival of 15 months after maximum resection of the tumor followed by the current standard radiochemotherapy.^1^ Bevacizumab (Bev), a humanized monoclonal antibody targeting vascular endothelial growth factor (VEGF), is commonly used for the treatment of recurrent GBM.^2^, ^3^ Bev often reduces gadolinium‐enhanced tumors and peritumoral edema on magnetic resonance imaging (MRI) in GBM and remarkably improves the clinical symptoms, including the Karnofsky performance status (KPS) of patients with GBM. However, some patients do not benefit from the antiangiogenic effect of Bev. Resistance to Bev therapy is thought to occur by various mechanisms including hypoxia‐induced activation of alternate angiogenic factors^4^, ^5^; vessel co‐option, the process whereby tumors utilize normal brain vessels to obtain oxygen and nutrients^6^; and inherent insensitivity of tumor vessels to VEGF signaling.^7^, ^8^ However, a predictive biomarker for responsiveness to Bev therapy has not been identified. Previous studies reported that Bev treatment at the time of recurrence improves clinical symptoms and prolongs progression‐free survival time (PFST), although Bev has no significant effect on overall survival (OS).^9^, ^10^ These reports suggest the need to stratify GBM patients with a reliable predictor according to responsiveness to Bev therapy. We previously reported that GBM expressing high CD44 in the tumor periphery shows a highly invasive phenotype on MRI and is associated with worse outcomes than GBM expressing low CD44.^11^ Among eight patients expressing high CD44, three patients who expressed CD44 at a very high level in the tumor periphery showed early tumor progression within 2 months after Bev therapy at tumor recurrence. These results suggested that high expression of CD44 in the tumor periphery may be related to resistance to Bev therapy in GBM.

Here, we analyzed patterns of responsiveness to Bev therapy and investigated the relationship between responsiveness to Bev and expression of CD44 and VEGF. In addition, to elucidate the roles of CD44 in anti‐VEGF therapy, we performed in vitro and in vivo studies using glioma stem‐like cells (GSCs) and a GSC‐transplanted mouse xenograft model, respectively. Identification of biomarkers predicting responsiveness to Bev may be useful for selecting patients for Bev treatment. Also, determination of the reason for ineffectiveness of Bev will provide important information for development of a novel therapeutic method for patients with Bev‐refractory GBM.

## 2 MATERIALS AND METHODS

All procedures performed in studies involving human participants were in accordance with the ethical standards of the institutional and/or national research committee and the 1964 Helsinki Declaration and its later amendments or comparable ethical standards. The present study was approved by the Ethics Committee for Clinical Research of Ehime University Hospital (no. 2006026).

### 2.1 Patients and study design

Fifty‐eight of 78 patients who were treated according to the same treatment protocol for primary GBM at Ehime University Hospital between April 2014 and September 2020 had tumor recurrence or progression. Among these 58 GBM patients, 22 patients who received only Bev as treatment against the first recurrence of tumors were enrolled in the present study. Other requirements for enrollment included a KPS score ≥50; methionine (Met)‐positron emission tomography (PET)‐certified recurrence of the tumor; adequate hepatic, renal, and cardiopulmonary function; and normal hematology. The recurrence of the tumor was confirmed by positive uptake of Met at a tumor/contralateral normal tissue ratio not less than 1.4 on Met‐PET in addition to tumor enhancement on gadolinium‐enhanced MRI.^12^ Informed consent was obtained from all individual participants enrolled in the study approved by the Ethics Committee for Clinical Research of Ehime University Hospital (no. 2006026). Our treatment protocol for primary GBM consists of maximal tumor resection with the aid of multimodal navigation systems including fens‐post echo‐guided navigation and 5‐aminolevulinic acid (ALA) fluorescence‐guided surgery, followed by radiotherapy (60 Gy) and chemotherapy with temozolomide in accordance with the Stupp protocol.^13^ As antiangiogenic therapy for recurrent tumors, 10 mg/kg Bev was intravenously administered every 2 weeks if toxicity was acceptable until tumor progression was confirmed. Progression of tumors after Bev therapy was determined by confirming that patients showed worsening of neurological symptoms and that imaging studies disclosed an increase in the high‐intensity area on fluid attenuated inversion recovery MRI and/or positive uptake of Met at a tumor/contralateral normal tissue ratio not less than 1.4 on Met‐PET. In the present study, no patient discontinued Bev therapy due to adverse effects. In all 22 patients, tumor tissue samples from two different sites, the tumor core and tumor periphery, were obtained using the previously described procedure^11^ and frozen and stored at −80°C until use. To find factors related to responsiveness to Bev therapy in recurrent GBMs, clinical features including age, sex, KPS at recurrence, and extent of resection (EOR) in the primary operation were examined for their associations to responsiveness to Bev. Other features, including the status of methylation of the *O (6)‐methylguanine‐DNA methyltransferase* promoter, *isocitrate dehydrogenase 1* (*IDH1*) mutation, and Ki‐67 staining index were evaluated with immunohistochemical analysis and also examined for their associations to responsiveness to Bev. In addition, to analyze the relationship between the effects of Bev and the invasiveness and proliferative activities of the tumors, expression of CD44 and VEGF in the tumor periphery, which are related to the invasive or proliferative type of GBM,^11^ was examined.

We also elucidated the mechanism of responsiveness to Bev by investigating the relationship between CD44 and VEGF and the roles of CD44 in Bev therapy with in vitro and in vivo studies.

### 2.2 RNA isolation and quantitative real‐time RT‐PCR

Total RNA was extracted from tissues of each tumor sample (core and periphery) and GSCs using ISOGEN (Nippon Gene) according to the manufacturer’s instructions. cDNA was synthesized using ReverTra Ace qPCR RT Master Mix with a gDNA remover kit (Toyobo). Quantitative real‐time RT‐PCR (qPCR) analysis was performed using Fast Start Universal SYBR Green Master Mix (Roche Diagnostic Japan) with an MJ mini instrument (BioRad). All gene‐specific mRNA expression values were normalized to the expression level of the housekeeping (reference) gene, glyceraldehyde‐3‐phosphate dehydrogenase (GAPDH). Quantification of gene expression was performed using ΔCt values, wherein ΔCt was defined as the difference between the target and reference gene Ct values. All primer sequences are listed in Table S1.

### 2.3 GSC culture

Three human GSC lines, GSC‐HI, GSC‐LI, and GDC40, were used in the present study. GSC‐HI (previously designated SFC‐2) was established from the primary cell culture of tissues surgically obtained from the tumor periphery of an invasive‐type GBM expressing high CD44.^11^ GSC‐LI was established in the same way from a moderately invasive GBM expressing lower levels of CD44. Details of the culture methods were previously described.^11^ GDC40 (glioblastoma‐derived cells) was isolated from GBM specimens using a serum‐free suspension culture method as previously described.^14^, ^15^ These three GSC lines were cultured in serum‐free DMEM/Ham’s F‐12 medium (Wako) containing 10 μg/ml insulin (Wako), 10 nmol/L recombinant human basic fibroblast growth factor, 10 nmol/L recombinant human epidermal growth factor, 5 μmol/L heparin, N2 supplement (Wako), GlutaMAX Supplement (GIBCO), and penicillin/streptomycin/amphotericin B mixture (neural stem cell medium). Growth factors were purchased from PeproTech. The stemness of these GSC lines was confirmed by evaluating their sphere‐forming ability before every use.

### 2.4 Treatment of GSC lines with small interfering RNA or Bev

GSC lines were treated with VEGF small interfering RNA (siRNA) or Bev to examine the relationship between intracellular and extracellular VEGF and expression of CD44. Effects of silencing VEGF on CD44‐mediated invasive and migratory activities of GSC lines were also investigated.

The sequences of siRNAs for VEGF were as follows: Sense 5′‐GGAGUACCCUGAUGAGAUCdTdT‐3′, Antisense 5′‐GAUCUCAUCAGGGUACUCCdTdT‐3′. As a control for each siRNA, we used a corresponding random siRNA sequence (5′‐GCGCGCUUUGUAGGAUUCG dTdT‐3′). GSCs were transfected with the siRNA using Lipofectamine 3000 reagent (Invitrogen) according to the manufacturer’s instructions. After a 24‐h incubation of GSCs transfected with each siRNA, the culture medium was changed to remove the Lipofectamine, and subsequent experimentation was performed. In addition, GSCs were treated with 1 mg/ml Bev (Chugai Pharmaceutical Co., Ltd.), and expression of VEGF and CD44 was examined.

### 2.5 Western blot analysis

Cells grown on poly‐l‐lysine‐coated dishes were lysed using RIPA buffer solution. The lysates were electrophoresed, transferred onto nitrocellulose membranes, and immunoblotted with antibodies to β‐actin (1:1000; mouse monoclonal; Sigma), CD44 (1:250; mouse monoclonal; Cell Signaling Technology), or VEGF (1:50; rabbit polyclonal; Abcam). Following incubation with alkaline phosphatase–conjugated secondary antibody (Promega), immunoreactions were developed using nitro blue tetrazolium and 5‐bromo‐4‐chloro‐3‐indolyl phosphate.

### 2.6 Immunohistochemical analysis

Cultured cells were fixed with 4% paraformaldehyde, permeabilized, and blocked with bovine serum albumin (BSA)‐tris‐buffered saline containing 0.1% tween 20 (TBSt) for 30 min. The cells were then incubated in a humidified chamber overnight at 4°C with a mixture of two primary antibodies diluted in BSA‐TBSt: antibodies to CD44 (1:250, mouse monoclonal, Cell Signaling Technology) and VEGF (1:50, rabbit polyclonal, Abcam). The cells were then treated as described above. After washing with TBSt, sections were treated with DyLight 488‐labeled anti‐mouse and Cy3‐conjugated anti‐rabbit IgG secondary antibodies (1:1000, Jackson ImmunoResearch). Hoechst 33342 (Sigma‐Aldrich) was used for nuclear staining. The immunostained specimens were observed under a conventional microscope (BX52; Olympus).

Sections of mouse brain (from experiments described in Section 9 below) were deparaffinized in Histo‐Clear (Cosmo Bio), hydrated in a graded alcohol series, and subjected to heat‐activated antigen retrieval. After blocking endogenous peroxidase activity, the sections were incubated in a humidified chamber overnight at 4°C with monoclonal antibodies to CD44 (1:200, Cell Signaling Technology), Ki‐67 (1:200, Dako), and CD34 (1:200, Abcam) diluted in BSA‐TBSt. Subsequently, the sections were washed with TBSt and incubated with biotinylated secondary antibody for 1 h at room temperature. The reaction complexes were stained with diaminobenzidine and counterstained with hematoxylin.

### 2.7 Cell invasion and migration assays

The invasiveness of cultured GSCs was assessed with an in vitro assay using Falcon cell culture inserts (Becton Dickinson Biosciences) and a reconstituted basement membrane, Matrigel (Becton Dickinson Biosciences), as previously described.^16^, ^17^ Briefly, GSCs were suspended in DMEM containing 0.1% BSA and seeded onto the insert filters at a density of 5.0 × 10^4^ cells/insert. The insert was placed in the lower wells of the Falcon 24‐well plate containing 500 μl DMEM with 1% fetal bovine serum and incubated for 24 h at 37°C in normoxic conditions. GSC migration was assayed using the modified Boyden chamber method with 48‐well microchemotaxis chambers (Nucleopore), as previously described.^18^, ^19^ GSCs in DMEM containing 0.1% BSA (at a density of 1 × 10^4^ cells/ml) were placed in the upper well, and DMEM containing 1% fetal bovine serum was placed in the lower well. A polyvinylpyrrolidone‐free polycarbonate membrane with 8‐μm pores (EMD Millipore) was used. In both assays, cells on the upper membrane surface were mechanically removed. Cells that had invaded or migrated to the lower side of the membrane were fixed, stained with 0.1% crystal violet, and examined under a microscope (×400) to determine the number of cells in three random fields.

### 2.8 Construction and establishment of GDC40 cells stably expressing CD44

Human CD44 cDNA was prepared from a GSC‐HI cell cDNA pool with PCR (sense primer: 5′‐aattctccgaacgtg atggacaagttttggtggca‐3′, antisense primer: 5′‐tcctacaaagcgcgc tca gctaatcttcttgaacagccgccagccgctcac caccccaatcttcatgtcca‐3′), tagged with HiBiT‐tag (Promega) at the 3′ termini. The PCR fragment was cloned into the pCX4 GFP retroviral vector (GenBank: AB296083.1) using the infusion cloning method (TaKaRa‐Clontech, Japan) as stated in the manufacturer’s instructions. pCX4GFP‐human CD44‐HiBiT and empty pCX4GFP vectors were packaged into retrovirus particles by co‐transfecting with pGP and pE‐ampho (TaKaRa retrovirus packaging kit) to 293T cells. Forty‐eight hours after the transfection, viral supernatants were collected and applied to GDC40 cells. After several passages, infection‐positive cells were collected by cell sorting with a FACS Aria (Beckton‐Dickenson) according to green fluorescence. pCX4GFP‐infected GDC40 was designated GDC40 (GFP), and CD44‐forced expressed GDC40 was designated GDC40(GFP)CD44. CD44 expression was confirmed with the LgBit‐mediated HiBiT‐tag detection system (Promega) as stated in the manufacturer’s instructions.

### 2.9 In vivo xenograft experiment with Bev administration

GSC‐HI, GSC‐LI, GDC40(GFP), and GDC(GFP)CD44 cells (1 × 10^6^) were suspended in 5 µl Matrigel and injected into the brain of 6‐week‐old male NOD/SCID mice that had been anesthetized intraperitoneally with a mixture of medetomidine (0.75 mg/kg), midazolam (4 mg/kg), and butorphanol tartrate (5 mg/kg). MRI was performed to confirm tumorigenesis 2 weeks after tumor implantation. Subsequently, Bev (6 mg/kg in NOD/SCID mice) was administered three times per week intraperitoneally, starting on day 15 after tumor cell implantation. We assessed the survival time of the model mice using Kaplan–Meier survival analysis.

### 2.10 Statistical analysis

Values are expressed as the mean ± standard deviation, and the data were compared using the Student’s *t* test (unpaired). Kaplan–Meier plots were generated to estimate OS from initiation of Bev therapy until death and from initial treatment for primary tumor until death. Patients alive at the last follow‐up were censored in the analysis. The log‐rank test was performed to assess the statistical significance of differences between groups. Significance was set at *p* < 0.05. All analyses were performed using Office Excel 2016 software (Microsoft®).

## 3 RESULTS

### 3.1 Patient characteristics

Patient characteristics are summarized in Table 1. The mean age of the 22 patients (17 males and 5 females) at tumor recurrence was 63.7 years (range 37–80 years), and they presented with a median KPS score of 70 (range 50–90). All 22 patients had no mutation in *IDH1*. The status of methylation of the *O (6)‐methylguanine‐DNA methyltransferase* promoter and the Ki‐67 staining index of the primary tumors are also summarized in Table 1. The EOR was evaluated with volumetric analysis on MRI before and after surgery as previously described.^20^ Gross total resection (100% resection), subtotal resection (95–100%), and partial resection (<95%) were achieved in 12 (70.6%), one (5.9%), and four patients (23.5%), respectively.

**TABLE 1:** Patient characteristics before Bev therapy and values of expression of CD44 and VEGF in the tumor periphery (P/C ratio for CD44), outcome after Bev therapy and sensitivity to Bev in 22 patients with GBM

Patient no.	Age (y) Sex	MGMT (m)	Ki‐67 LI (%)	EOR	KPS (%)	CD44	VEGF	PFST	OST	OS (M)	Outcome	Response type
						(P/C ratio)	(Periphery)	on Bev (M)	on Bev(M)			
1	63F	−	33	GTR	80	1.3	32.6	12	13	59	D	S
2	62M	−	17	STR	70	14.1	2.5	3	3	17	D	M
3	66M	+	45	GTR	70	18.4	0.7	2	9	16	D	R
4	53M	+	7	GTR	80	9.8	1	5	24	33	D	M
5	61F	+	42	GTR	80	0.65	4.25	6.5	18	26	D	M
6	79M	+	12	GTR	50	32.8	1.8	2	9	18	D	R
7	75M	−	13	GTR	50	18	0.9	1	3	14	D	R
8	44M	−	51	GTR	80	0.85	8.6	13	23+	47+	A	S
9	80M	−	15	PR	60	0.27	16.1	3.5	8	9	D	M
10	53M	+	30	GTR	80	3.2	1.51	3	11	13	D	M
11	69M	−	30	PR	90	1.03	11.5	23	26	28	D	S
12	53F	+	60	GTR	80	3.2	4.54	2	2	8	D	R
13	64M	−	40	GTR	70	3.2	3.31	3	9	16	D	M
14	64M	−	25	PR	70	13.05	18.06	6	11	14	D	M
15	60M	+	40	PR	70	7.3	5.26	11	16	20	D	M
16	52F	−	40	GTR	80	1.4	9.17	16	26+	34+	A	S
17	76M	+	30	GTR	70	0.83	12.3	20	24	25	D	S
18	74M	−	50	GTR	90	19.1	3.63	1.5	5	21	D	R
19	77M	−	15	GTR	90	0.15	3.88	10.5	13	19	D	M
20	72M	−	30	GTR	90	1.19	0.08	5	6+	22+	A	M
21	58F	−	40	GTR	90	2.55	1.31	3.5	4+	17+	A	M
22	37M	−	55	PR	90	9.84	7.6	11.5	12+	14+	A	M

+, survival time in the patient alive at the last follow‐up; A, alive; Bev, bevacizumab; D, dead; EOR, extent of resection; F, female; GTR, gross total resection; KPS, karnofsky performance status; LI, labeling index; M, male; M, moderately sensitive to Bev; M, month; MGMT(m), methylation of O(6)‐methylguanine‐DNA methyltransferase; No., number; OS, overall survival; OST, overall survival time; P/C ratio, periphery/core ratio; PFST, progression‐free survival time; PR, partial resection; R, resistant to Bev; S, sensitive to Bev.**;** STR, subtotal resection; VEGF, vascular endothelial growth factor.

To evaluate the antitumor effects of Bev therapy, responsiveness to Bev was classified into three types based on the period from initiation of Bev therapy until tumor progression (PFST). These included resistant (R‐type) (<3 months): five patients, moderately sensitive (M‐type) (≥3 months to <12 months): 12 patients, highly sensitive (S‐type) (≥12 months): five patients. PFST <3 months corresponds to treatment of not more than six cycles of Bev therapy, and PFST ≥12 months corresponds to treatment not less than 24 cycles of Bev therapy (Table 1). No significant difference among the three types was found in age, sex, KPS, or EOR.

### 3.2 mRNA expression of CD44 and VEGF in the tumor tissues of 17 GBMs

**FIGURE 1:**
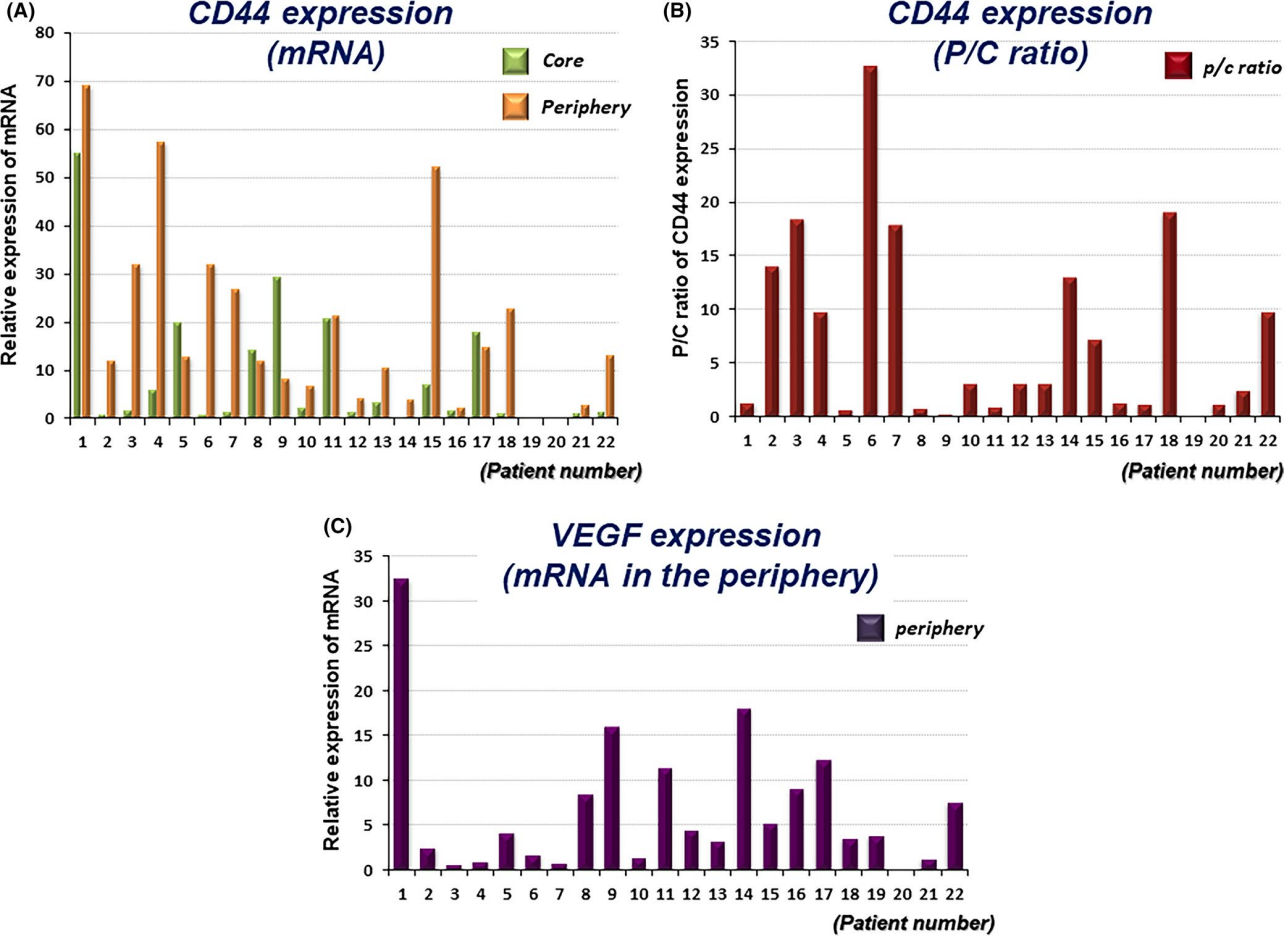
Expression of the stem cell marker, CD44, and the related molecule, vascular endothelial growth factor (VEGF), in 22 GBM patients. (A) mRNA expression of CD44 in the tumor core and periphery was determined with qRT‐PCR. The values are relative expression of mRNA normalized to GAPDH. (B) Expression of CD44 is shown as the P/C ratio. The P/C ratio of CD44 was calculated by dividing the amount of mRNA expression of CD44 in the tumor periphery by that in the core for each patient. (C) Expression of the stem cell‐related marker, VEGF. mRNA expression of VEGF in the tumor periphery was determined with qRT‐PCR. The values are relative expression of mRNA normalized to GAPDH

We analyzed the mRNA expression of the stem cell marker, CD44, and the stem cell‐related molecule, VEGF, in the tumor tissues from two different sites, the tumor core and tumor periphery of 22 GBM patients (Figure 1A). To evaluate the degree in expression of CD44 and VEGF in the tumor periphery, the periphery/core (P/C) ratio, which was obtained by calculating the ratio of mRNA expression of these molecules in the tumor periphery to that in the tumor core, was adopted for CD44, and the amount of mRNA was used for VEGF as described in our previous study^11^ (Figure 1B,C) (Table 1).

### 3.3 Relationship between CD44/VEGF expression and responsiveness to Bev, and prognostic outcome of the patients

**FIGURE 2:**
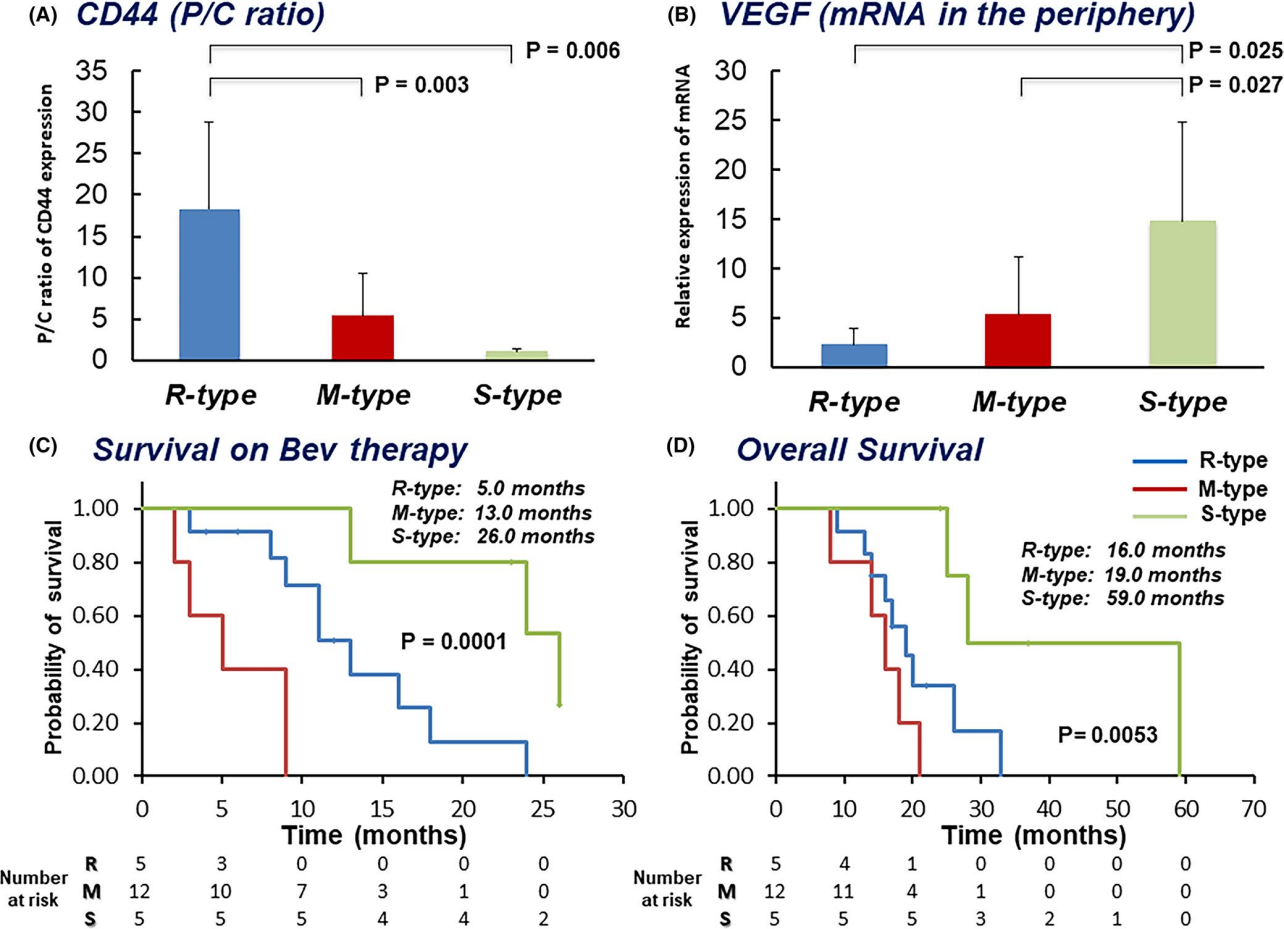
Expression of CD44 and VEGF in three types of Bev response and survival curves generated from patients with initial treatment for primary tumors and those with Bev therapy for recurrent tumors. (A) Relationship between the P/C ratio for CD44 expression and three types of Bev response. The R‐type showed the highest value for the P/C ratio of CD44, whereas the S‐type showed the lowest (R‐type vs S‐type, *p* = 0.006; R‐type vs M‐type, *p* = 0.003). (B) Relationship between VEGF expression in the tumor periphery and three types of Bev response. The S‐type showed significantly higher expression of VEGF than both the R‐type and the M‐type (S‐type vs R‐type, *p* = 0.025; S‐type vs M‐type, *p* = 0.027). (C) Kaplan–Meier survival curves of patients given Bev therapy to treat recurrence of the tumor (from initiation of Bev therapy until death). The S‐type showed significantly longer survival than the R‐type and M‐type (*p* = 0.0001). (D) Kaplan–Meier survival curves of patients with initial treatment for primary tumors (from surgical resection until death). The S‐type showed significantly longer survival than the R‐type and M‐type (*p* = 0.0053)

The mean P/C ratio of CD44 expression of the R‐type was 18.3, that of the M‐type was 5.44, and that of the S‐type was 1.08, demonstrating that the R‐type had the highest P/C ratio of CD44 expression among the three types. The S‐type showed the lowest P/C ratio of CD44 (R‐type vs S‐type, *p* = 0.006, R‐type vs M‐type, *p* = 0.003) (Figure 2A). The cut‐off value for the P/C ratio of CD44 for the R‐type was 18.0 (sensitivity 80%, specificity 100%). The S‐type showed the highest expression of VEGF among the three types, and the R‐type expressed the lowest level (S‐type vs M‐Type, *p* = 0.027, S‐type vs R‐type, *p* = 0.025) (Figure 2B). The S‐type showed the longest OS time for both the first recurrence and after treatment of the primary tumor among the three types. In contrast, the R‐type presented with the worst OS, both for treatment for the recurrence and the primary tumor (Figure 2C,D).

### 3.4 Inhibition of VEGF promotes CD44 expression in GSCs and enhances invasion and migration of GSCs

**FIGURE 3:**
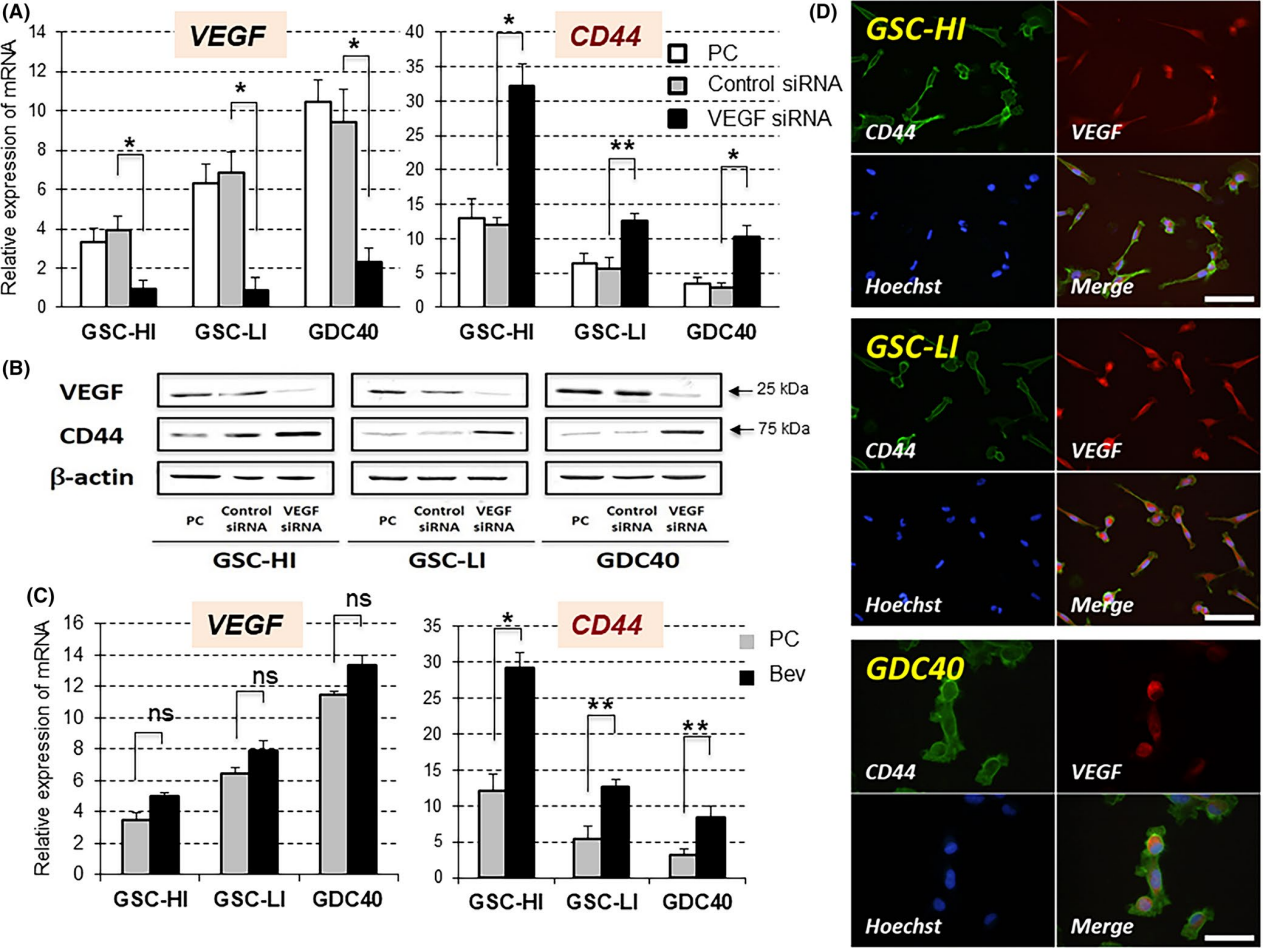
Inhibitory effects of VEGF on CD44 expression in GSC lines. (A) VEGF siRNA significantly inhibited the expression of VEGF in all three GSC lines. Inhibition of VEGF significantly upregulated the expression of CD44 in all GSC lines. PC: control *p < 0.001, **p < 0.005. (B) Western blot analysis demonstrating markedly increased expression of CD44 protein following silencing of VEGF with siRNA in all GSC lines. (C) Inhibition of VEGF with Bev did not affect the expression of VEGF but significantly upregulated the expression of CD44 in all GSC lines. *p < 0.001, **p < 0.005, ns: not significant. (D) Double‐labeling immunofluorescence demonstrating co‐localization of CD44 (green) and VEGF (red) in three GSC lines. Cell nuclei are labeled with Hoechst (blue) (scale bar: 100 μm)

As CD44 and VEGF in the tumor periphery of GBM tended to be expressed in a mutually exclusive manner in terms of responsiveness to Bev therapy, we investigated whether a relationship was present between VEGF and CD44 using three GSC lines. The features of the GSC lines are summarized in Figure S1. Silencing of *VEGF* using *VEGF* siRNA significantly up‐regulated both the mRNA and protein expression of CD44 in all three GSC lines (Figure 3A,B). Treatment of GSC lines with Bev also increased the expression of CD44 in all cell lines (Figure 3C). Double‐labeling immunofluorescence demonstrated that CD44 and VEGF were co‐localized in the same tumor cells (Figure 3D). Consequently, secreted VEGF may suppress the expression of CD44 in GSCs in an autocrine manner.

**FIGURE 4:**
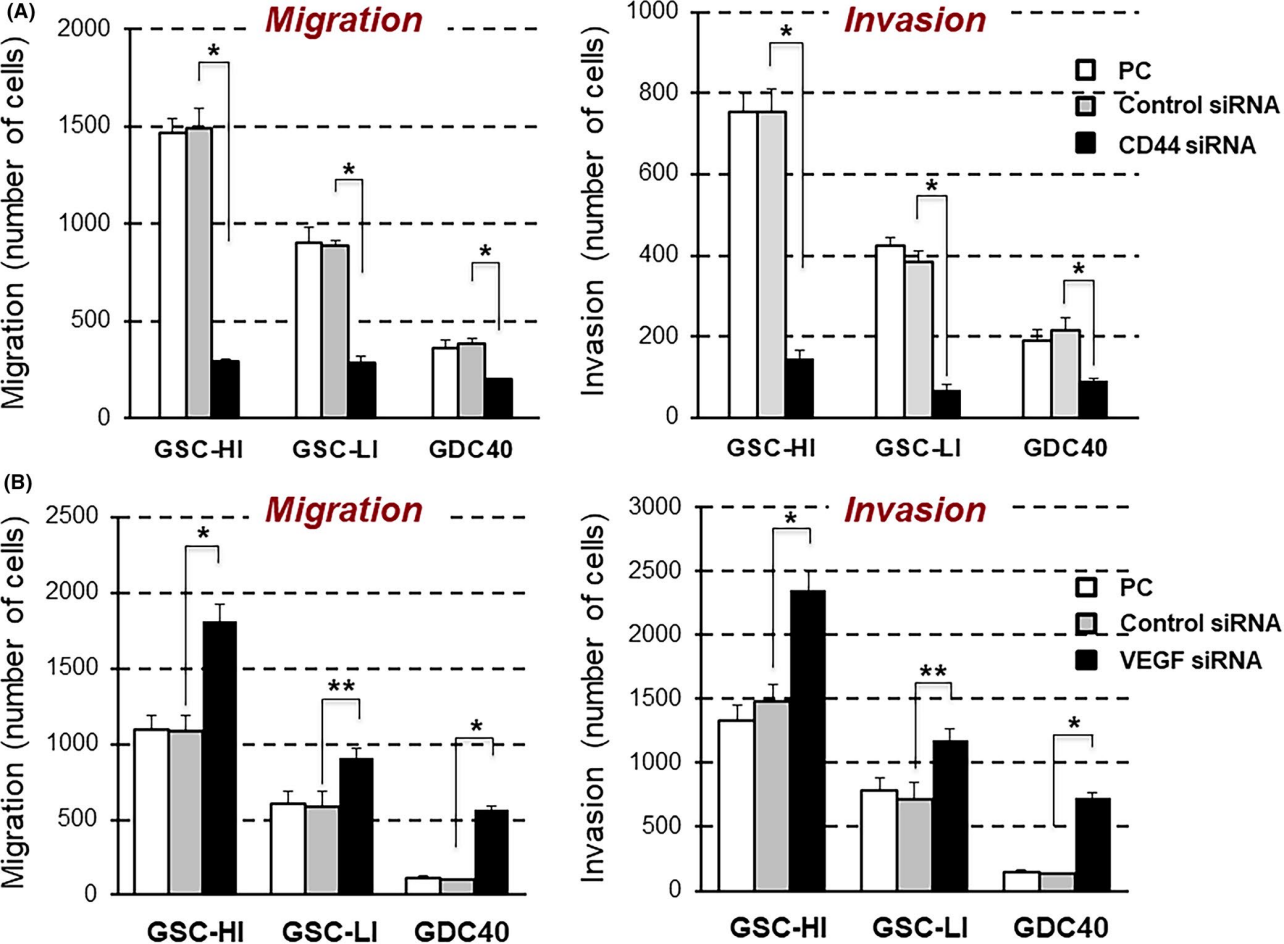
Inhibitory effects of VEGF on invasive/migratory activities of GSC lines. (A) High migratory and high invasive activities of GSC lines were significantly inhibited by treatment with CD44 siRNA. (B) Inhibition of VEGF with siRNA significantly enhanced migration and invasion in all GSC lines. *p < 0.001, **p < 0.05

The three GSC lines showed much higher activities of invasion and migration than the nonstem parent cells from which each GSC line was established. The degree of invasion and migration of GSC lines was dependent on the level of CD44 expression in these cell lines, and the activities were markedly inhibited by silencing CD44 with siRNA (Figure 4A). GSC‐HI cells expressed the highest level of CD44 and had the highest invasion and migration of the tumor cells, whereas GDC40 cells expressed the lowest level of CD44 and showed the lowest activities of invasion and migration. GSC‐LI cells expressed moderate levels of CD44 and showed intermediate activities of invasion and migration among the three lines. These activities of invasion and migration of the GSC lines were significantly enhanced by VEGF knockdown with siRNA (Figure 4B).

### 3.5 In vivo effects of Bev on survival of mice transplanted with GSC lines and GSCs with CD44 overexpression

**FIGURE 5:**
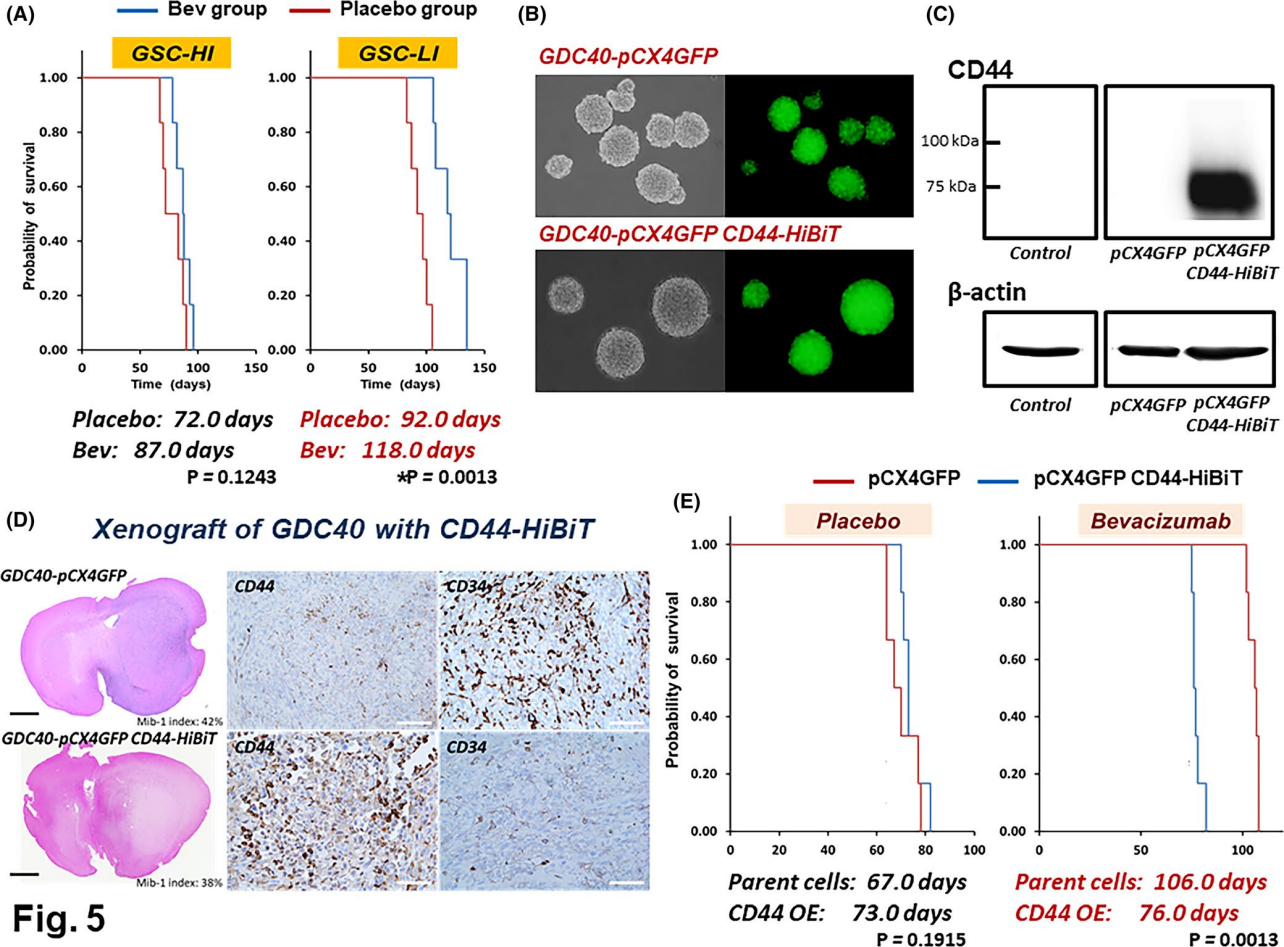
Effects of CD44 on the survival of mice with xenografted GSCs. (A) Kaplan–Meier survival curves of GSC‐transplanted mice with and without Bev treatment. The median OS times of the mice transplanted with GSC‐HI (high expression of CD44) and GSC‐LI (low expression of CD44) with no treatment were 72.0 and 92.0 days, respectively. On the contrary, when mice were treated with Bev, the median OS times of mice transplanted with GSC‐HI and GSC‐LI were 87.0 and 118.0 days, respectively. Only mice transplanted with GSC‐LI showed significantly longer survival with Bev therapy than the control (*p* = 0.0013). (B) The sphere‐forming ability of GDC40 cells infected with the constructed pCX4 GFP, in which CD44 tagged with the HiBiT‐tag was overexpressed, remained unimpaired. (C) Western blot demonstrating expression of a large amount of CD44 protein by pCX4GFP‐GDC40‐CD44‐HiBiT. (D) Histology of mouse brains transplanted with GDC40 cells with pCX4GFP and GDC40 cells with pCX4GFP‐CD44‐HiBiT showed that GDC40 cells with CD44‐HiBiT produced an indistinct margin of the tumor mass and diffuse infiltration into the surrounding brain compared with GDC40 cells with pCX4GFP (upper panel) (scale bar: 500 μm). Immunohistochemistry revealed that GDC40 cells with CD44‐HiBiT expressed much more CD44 but much less CD34 than control GDC40 cells. No difference was seen in the Mib‐1 (Ki‐67) proliferative index between the two (lower panel) (scale bar: 100 μm). (E) Kaplan–Meier survival curves for assessing the effects of Bev on mice transplanted with GDC40 cells with pCX4GFP and pCX4GFP‐CD44‐HiBiT. Mice transplanted with GDC40 cells with pCX4GFP showed significantly longer survival following treatment with Bev compared with placebo (no Bev) (Bev+: 106.0 days vs Bev−: 67.0 days, *p* = 0.0013). In contrast, mice transplanted with GDC40 cells with pCX4GFP‐CD44‐HiBiT did not show such a beneficial survival effect of Bev (Bev+: 76.0 days vs Bev−: 73.0 days, *p* = 0.1915)

The median OS times in GSC‐HI and GSC‐LI‐transplanted mice were 72 and 92 days, respectively. On the contrary, after treatment with Bev, the median OS times in GSC‐HI and GSC‐LI‐transplanted mice were 87.0 and 118.0 days, respectively. GSC‐HI‐transplanted mice did not show a significant survival benefit from Bev therapy (*p* = 0.1243), whereas GSC‐LI‐transplanted mice showed significantly longer survival time following treatment with Bev compared to no treatment (placebo) (*p* = 0.0013) (Figure 5A). Effects of Bev on CD44‐overexpressed GSCs that were transplanted into mouse brain were investigated using NOD/SCID mice transplanted with GDC40(GFP) with or without CD44‐HiBiT (Figure 5B). GDC40 with CD44 overexpression, designated GDC(GFP)CD44, produced large amounts of CD44 protein (Figure 5C). Transplantation of GDC40(GFP)CD44 into the brains of NOD‐SCID mice generated a much more diffusely extended tumor mass with an indistinct tumor margin compared with that of GDC40(GFP), which showed a relatively demarcated margin (Figure 5D). Immunohistochemistry revealed that the tumor generated from the transplant of GDC40(GFP)CD44 expressed CD44 in almost all tumor cells, whereas CD34‐positive neovascularization was very low compared with the tumor from the transplant of GDC40(GFP) (Figure 5D, middle and right panels). The median OS times of mice with tumors generated by transplants of GDC40(GFP) and GDC40(GFP)CD44 were 67.0 and 73.0 days, respectively, showing no significant difference in OS times between the two groups (*p* = 0.1915). On the contrary, when Bev was administered, median OS times of mice with transplants of GDC40(GFP) and GDC40(GFP)CD44 were 106.0 and 76.0 days, respectively. In the mice transplanted with GDC40 expressing low CD44, treatment with Bev significantly prolonged the survival time compared to no Bev treatment, but the mice transplanted with GDC40 overexpressing CD44 did not obtain a survival benefit when treated with Bev (*p* = 0.0013) (Figure 5E).

## 4 DISCUSSION

Antiangiogenic therapy with Bev has become common for the treatment of recurrent GBM, but its benefit for longer survival of patients is limited. To improve the prognostic outcome with Bev therapy, the reason for the difference in responsiveness to Bev needs to be determined. To date, several studies seeking to identify predictive biomarkers for responsiveness to Bev in GBM have been conducted, but reliable biomarkers remain unknown. Manneh Kopp et al^21^ reported no significant correlation between the clinical outcome in patients with recurrent GBM treated with Bev and histopathological parameters such as the Ki‐67 labeling index, various molecules including c‐Met, HIF‐1, and VEGFA, and neuroradiological parameters. Hovinga et al^22^ recently analyzed time to progression in GBM patients treated with Bev and reported that epidermal growth factor receptor amplification and the classical subtype are associated with a poor response to Bev in recurrent GBM. Here, we demonstrated that the P/C ratio of CD44 expression in GBM was significantly correlated with responsiveness to Bev for the treatment of recurrent GBM. Pearson’s correlation analysis in the present patients with GBM demonstrated that the P/C ratio of CD44 was significantly more correlated with PFST on Bev therapy than mRNA expression of CD44 in the tumor periphery, whereas mRNA expression of VEGF in the tumor periphery showed significantly a much better correlation with PFST than the P/C ratio of VEGF (Figure S2).These results suggest that the present evaluation of the degree of CD44 and VEGF expression is more appropriate than other assessments. As described in our previous study, the P/C ratio of CD44 may be thought to represent the degree of CD44 expression that is up‐regulated on GSCs residing in the specific microenvironment of the tumor periphery in GBM.^11^ GBM patients with short PFST (<3 months) after initiation of Bev therapy (R‐type) showed a high P/C ratio of CD44, whereas patients with longer PFST (≥12 months) after Bev therapy (S‐type) showed a low P/C ratio of CD44. In contrast, S‐type tumors expressed VEGF at the highest level in the tumor periphery of GBM, and R‐type tumors expressed VEGF at the lowest level among the three types. These results suggest that the responsiveness of recurrent tumors to Bev therapy represents the biological nature of the primary tumors in the tumor periphery of GBM at the time of initial treatment. Thus, identification of a biomarker for responsiveness to Bev would allow selection of patients suitable for Bev therapy, even at the time of primary treatment for GBM. We previously reported that GBMs expressing high CD44 in the tumor periphery show a highly invasive phenotype on MRI and are associated with early tumor progression and worse prognosis compared with GBMs expressing low CD44, which represent a less invasive and highly proliferative phenotype on MRI.^11^ In the present study, patients with R‐type tumors expressing high CD44 in the tumor periphery of GBM showed the shortest survival time after Bev therapy, and the primary tumor showed a much more invasive type on MRI compared with patients with S‐type tumors (Figure S3).

CD44 is a nonkinase transmembrane glycoprotein that is expressed in several cell types including cancer stem cells.^23^ CD44 is a multifunctional molecule that binds to specific ligands and activates signaling pathways of various cellular processes, including promotion of cell proliferation, cell survival, cell motility, and modulation of angiogenesis.^24^, ^25^ Recently, we demonstrated that CD44 is a dual‐functional molecule that can promote either invasion or proliferation of GSCs according to the degree of hypoxia. CD44 expression is up‐regulated by severe hypoxia (1% O_2_) (manuscript submitted). How CD44 with such specific features participates in resistance to anti‐VEGF therapy is unknown.

Major mechanisms of resistance to anti‐VEGF therapy with Bev include activation of alternate pathways by pro‐angiogenic factors such as HGF, fibroblast growth factor, and Ang‐2^26^, ^27^; selection of malignant cells showing resistance to anti‐VEGF therapy‐induced hypoxia, leading to increased invasion and metastasis^7^, ^28^; and increased tumor vessels with inherently low sensitivity to VEGF inhibition.^29^, ^30^ In the present study, GBMs with higher CD44 and lower VEGF expression in the tumor periphery showed much more resistance to Bev therapy than GBMs with lower CD44 and higher VEGF expression in the tumor periphery.

Why are GBMs with high CD44 and low VEGF expression refractory to Bev, and what is the relationship between CD44 and VEGF during treatment with Bev for GBM? To answer these questions and clarify the role for CD44 in VEGF‐promoted angiogenesis, we investigated the inhibitory effects of VEGF on CD44 expression and tumor invasion using cultured GSC lines because GSCs in the tumor border are thought to be causal cells expressing activated CD44. In vitro inhibition of VEGF with siRNA significantly up‐regulated the expression of CD44 in all GSC lines. In vitro treatment with Bev also increased the expression of CD44 in all GSC lines. These results indicate that secreted VEGF outside tumor cells may regulate the expression of CD44 via the autocrine signaling pathway of VEGF/VEGFR‐2 on GSCs. Inhibition of VEGF significantly enhanced invasion and migration of GSC lines whose activities were inhibited by knockdown of *CD44*.

These results suggest that inhibiting VEGF by treatment with Bev in recurrent GBM may induce or promote higher invasion of tumor cells by enhancing CD44 expression. Several studies have shown that inhibition of angiogenesis may promote tumor cell invasiveness and formation of metastasis.^7^ Lu et al^31^ reported that VEGF blockade with Bev up‐regulates the receptor tyrosine kinase c‐Met activity in GBM cells, resulting in enhanced invasiveness of the cells via activation of the HGF/c‐Met pathway. McCarty reported that tumor cell proliferation and invasion are regulated by cross‐talk between the VEGF and HGF signaling pathways. VEGFR‐2 and c‐Met form heterodimeric complexes that regulate tumor cell growth and invasion. When c‐Met is upregulated by inhibition of VEGF with Bev, c‐Met forms homodimers to which HGF/SF binds and promotes tumor cell invasion.^32^ Also, the CD44 variant isoform, CD44v6, acts as a co‐receptor for the receptor tyrosine kinase, c‐Met, and promotes tumor cell invasion.^33^ We found that inhibition of VEGF activated not only CD44 expression, but also c‐Met expression in GSC lines (Figure S4). These results suggest that VEGF secreted from GSCs not only has an angiogenic effect on the endothelial cells of tumor vessels in a paracrine manner through VEGFR2 on endothelial cells, but also negatively regulates expression of CD44 and c‐Met in an autocrine manner through VEGFR2 on the tumor cells, thus, resulting in suppression of tumor invasion and migration when the tumor is proliferating due to the angiogenic activity of VEGF. In general, tumor cells do not proliferate when they migrate and vice versa.^34^, ^35^ Thus, these activities of VEGF in GSCs may recapitulate the biological nature of tumor cells.

We also tested the effects of CD44 on the survival of mice treated with Bev using an in vivo mouse xenograft model and transplantation of three GSC lines. Mice transplanted with GSC‐HI cells, which express high levels of CD44, did not show significantly prolonged survival time following treatment with Bev, whereas mice transplanted with GSC‐LI or GDC40 cells, which both express much lower levels of CD44 than GSC‐HI cells, showed much longer survival following treatment with Bev than the control. Furthermore, mice transplanted with GDC40 cells with CD44 overexpression did not show prolonged survival following Bev treatment. These in vivo studies indicate that GSCs with inherently high expression of CD44 may not be affected by the antitumor activity of Bev, resulting in early resistance to Bev.

In the present study, we classified the patients with recurrent GBM regarding responsiveness to Bev into three types according to the duration of Bev effectiveness. R‐type patients may be thought to have inherent resistance to Bev. Four of these patients expressed CD44 at a very high level (P/C ratio ≥18.0) and showed short OS time. These patients may have had a very poor prognosis beginning at the time of the initial treatment for the primary GBM including nonresponsiveness to any therapy including Bev. Furthermore, in the patients with high CD44 expression, treatment with Bev may make the outcome much worse. Among M‐type patients, those who show a high P/C ratio of CD44 expression may develop resistance to Bev relatively early after Bev therapy, resulting in a much more highly invasive tumor. Thus, high CD44 expression in the tumor periphery of GBM (cut‐off value for the P/C ratio for R‐type: 18.0) will become a useful biomarker for predicting the degree of responsiveness to Bev. Also, CD44 may serve as a therapeutic target for effectively inhibiting both tumor invasion and proliferation in GBM.

This study has some limitations. The present study was a retrospective analysis of a small number of patients because requirements included tumor tissues that were accurately taken from two different sites (periphery and core in GBM). In addition, only Bev was used for the treatment at first recurrence. Accordingly, the statistical analysis has some limitations. More extensive analysis with an increased number of patients and also a well‐designed prospective study will be required for a more definite conclusion and before application to clinical practice.

## 5 CONCLUSIONS

GBMs expressing CD44 at a much higher level in the tumor periphery than in the tumor core (high P/C ratio of CD44) were refractory to Bev therapy, and patients showed much shorter survival than when their tumors expressed a low P/C ratio of CD44. In vitro inhibition of VEGF by either siRNA or Bev activated the expression of CD44 in cultured GSCs and significantly promoted invasion and migration of the tumor cells. Bev treatment of mice transplanted with GSCs with low expression of CD44 induced significantly longer survival time than no Bev treatment. In contrast, Bev treatment of mice transplanted with GSCs with CD44 overexpression had no beneficial effect in prolonging survival of the mice. These results indicate that the ratio of CD44 expression in the tumor periphery to the tumor core will become a useful biomarker for predicting responsiveness to Bev. In addition, GBMs expressing high CD44 in the tumor periphery show enhanced expression of CD44 by inhibition of VEGF with Bev, leading to more invasive and aggressive tumors, and resulting in earlier progression and worse prognosis.

## CONFLICT OF INTEREST

The authors declare no potential conflicts of interest.

## Supporting information

Fig S1

Fig S2

Fig S3

Fig S4

Table S1

## Data Availability

The data that support the findings of this study are available from the corresponding author upon reasonable request.
