# Supplementary figures and images for: CD44 expression in the tumor periphery predicts the responsiveness to bevacizumab in the treatment of recurrent glioblastoma
Source: Cancer Med. 2021 Feb 5;10(6):2013–25. doi: 10.1002/cam4.3767 (PMC7957167; doi:10.1002/cam4.3767)

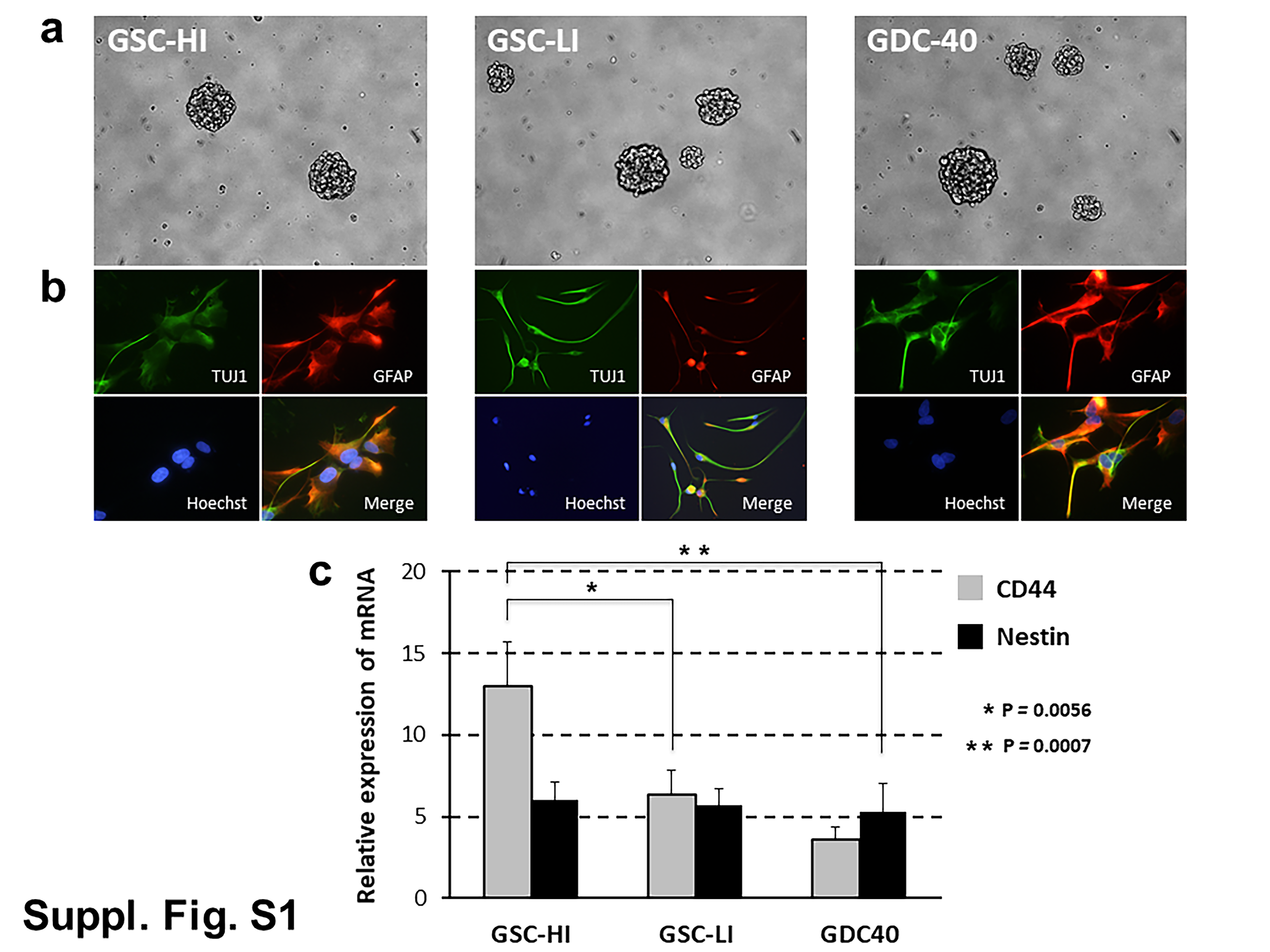

Supplement: Supplementary file 1 — Fig S1 [file CAM4-10-2013-s003.tif]

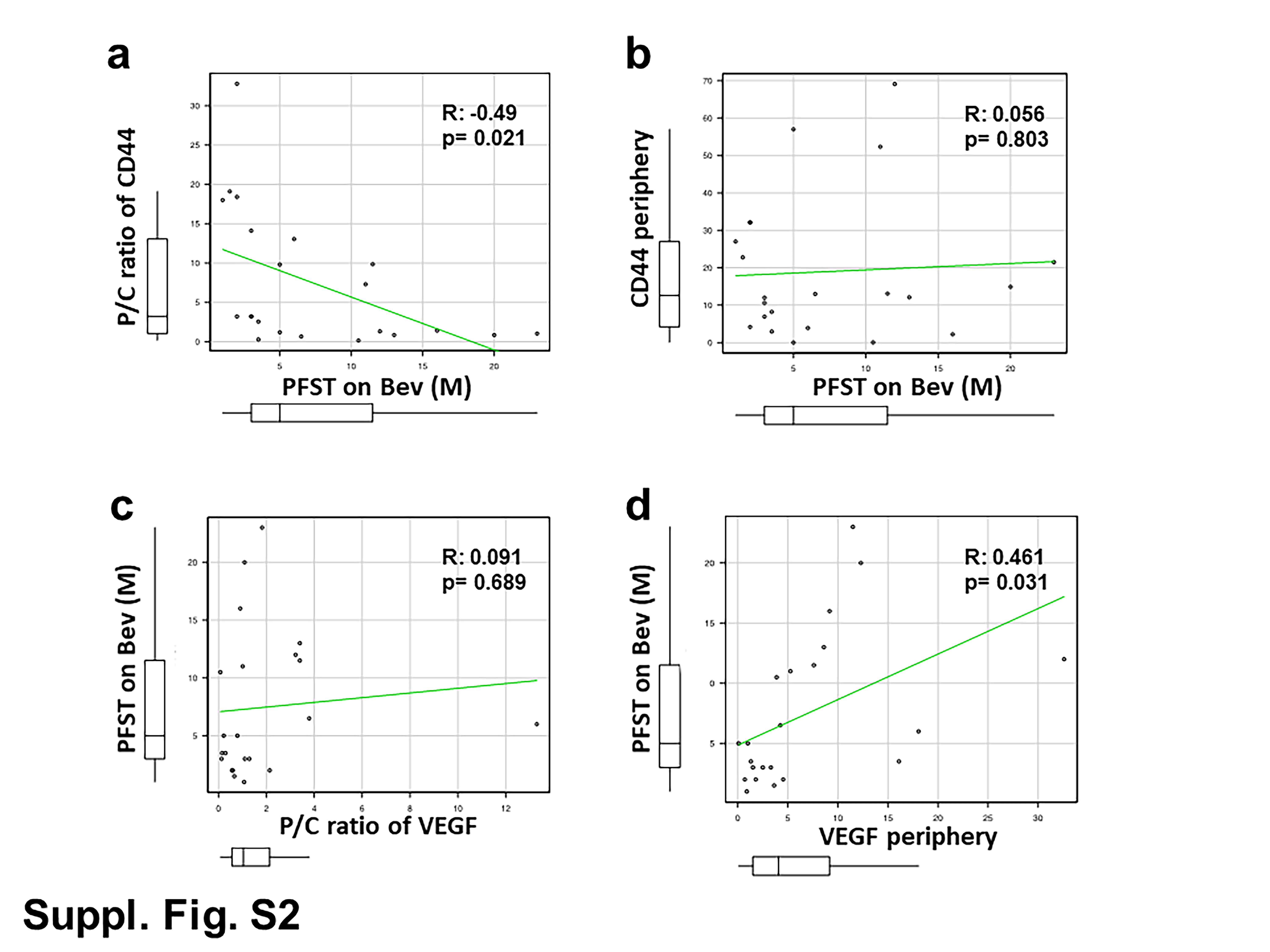

Supplement: Supplementary file 2 — Fig S2 [file CAM4-10-2013-s005.tif]

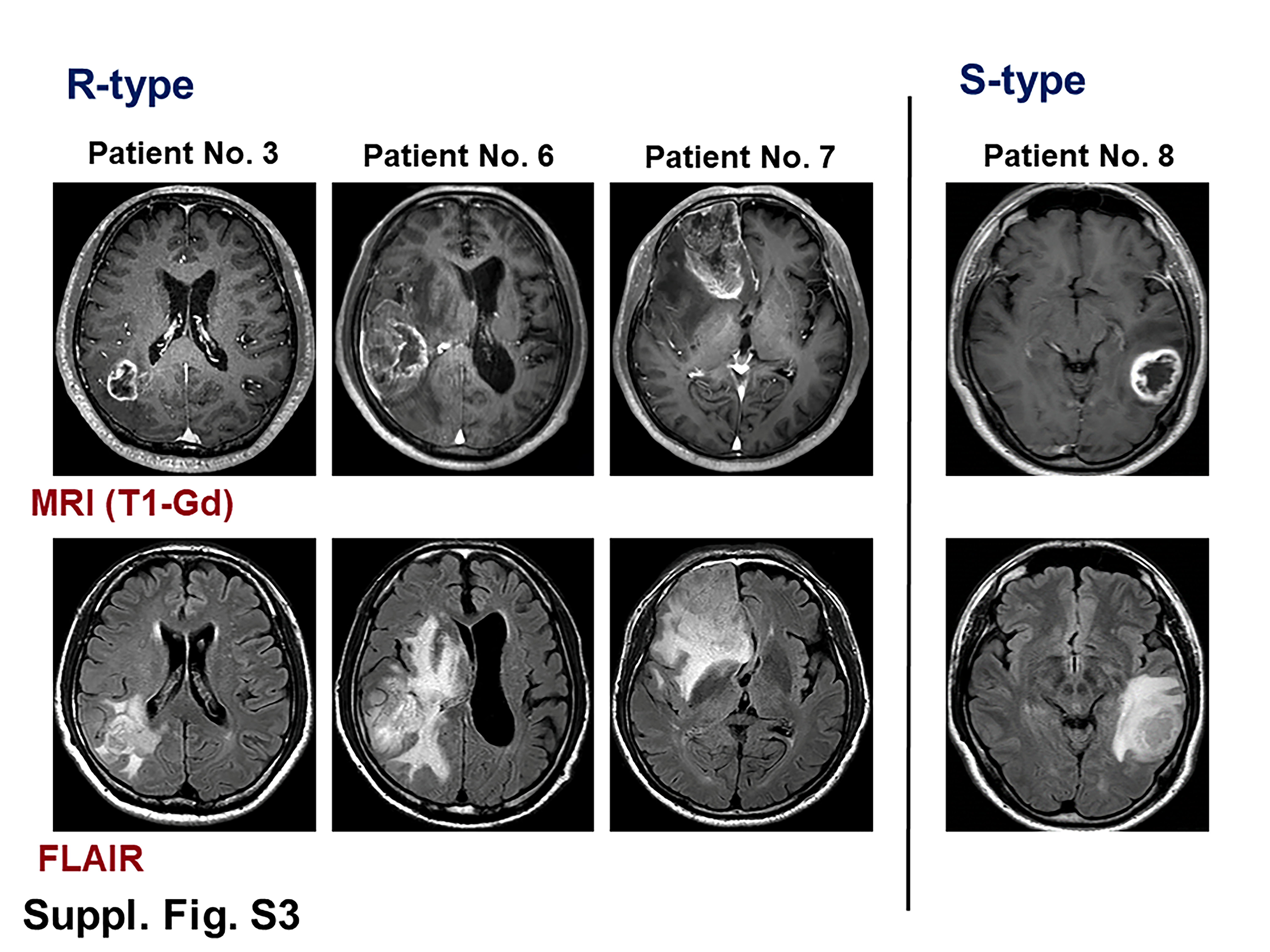

Supplement: Supplementary file 3 — Fig S3 [file CAM4-10-2013-s002.tif]

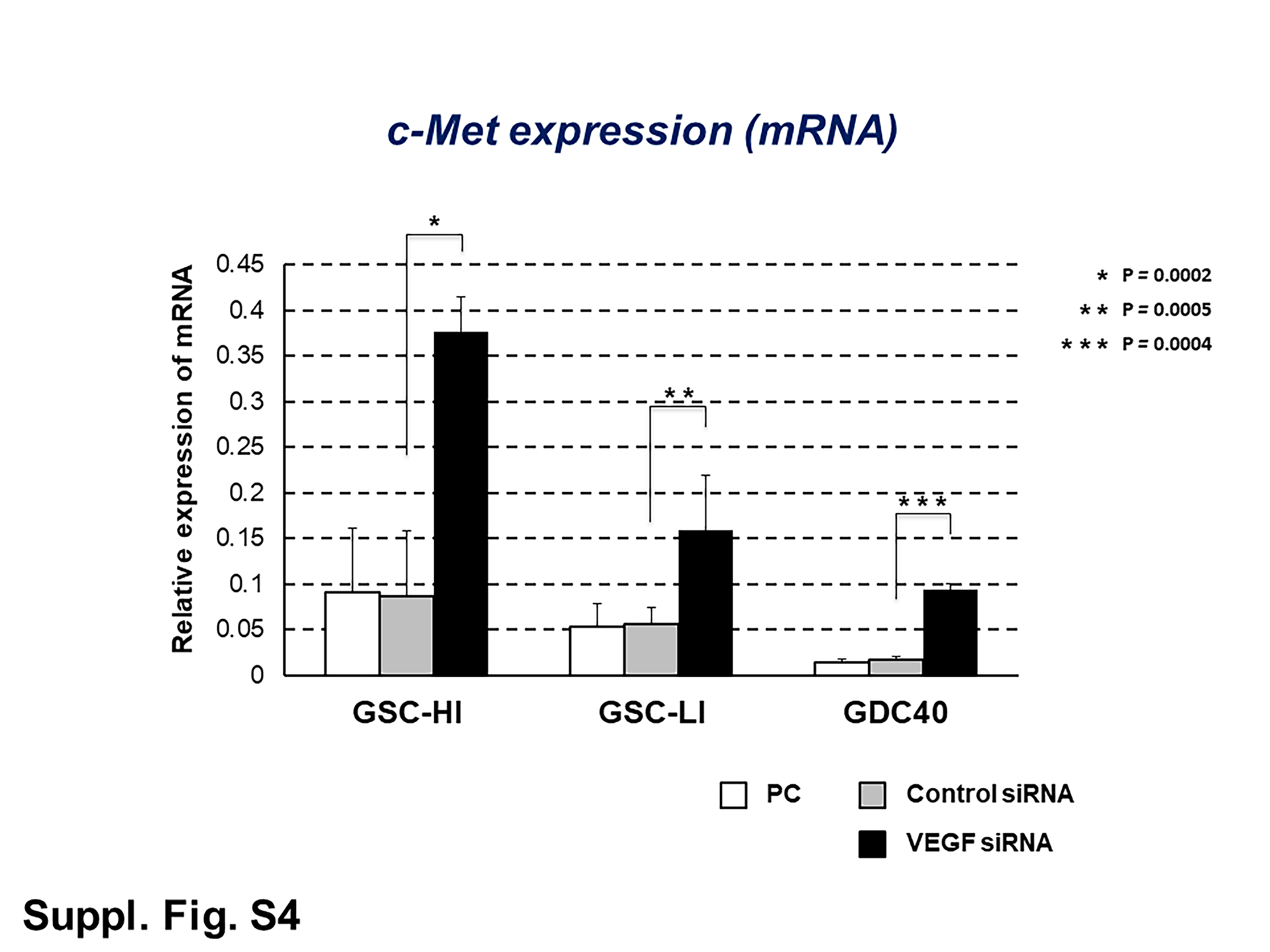

Supplement: Supplementary file 4 — Fig S4 [file CAM4-10-2013-s001.tif]
