# Supplementary material for: CD44 expression in the tumor periphery predicts the responsiveness to bevacizumab in the treatment of recurrent glioblastoma
Source: Cancer Med. 2021 Feb 5;10(6):2013–25. doi: 10.1002/cam4.3767 (PMC7957167; doi:10.1002/cam4.3767)
Supplement: Supplementary file 5 — Table S1 [file CAM4-10-2013-s004.pdf]

Suppl. Table S1: Oligonucleotide primers used for qRT-PCR

Gene	Sense/antisense
CD44	5’-AGAAGGTGTGGGCAGAAGAA-3’
	5’-AAATGCACCATTTCCTGAGA-3’
VEGF	5'- GGCGAAGAGAAGAGACACAT -3'
	5'- GAGGAAGGTCAACCACTCAC -3'
GAPDH	5'- CAGTCAGCCGCATCTTCTTT -3'
	5'- TGACGGTGCCATGGAATTTG -3'
